# The Genome Sequence of the Anthelmintic-Susceptible New Zealand *Haemonchus contortus*

**DOI:** 10.1093/gbe/evz141

**Published:** 2019-07-02

**Authors:** Nikola Palevich, Paul H Maclean, Abdul Baten, Richard W Scott, David M Leathwick

**Affiliations:** AgResearch Limited, Grasslands Research Centre, Palmerston North, New Zealand

**Keywords:** *Haemonchus contortus*, genome, helminth, parasite, anthelmintic-susceptible

## Abstract

Internal parasitic nematodes are a global animal health issue causing drastic losses in livestock. Here, we report a *H. contortus* representative draft genome to serve as a genetic resource to the scientific community and support future experimental research of molecular mechanisms in related parasites. A *de novo* hybrid assembly was generated from PCR-free whole genome sequence data, resulting in a chromosome-level assembly that is 465 Mb in size encoding 22,341 genes. The genome sequence presented here is consistent with the genome architecture of the existing *Haemonchus* species and is a valuable resource for future studies regarding population genetic structures of parasitic nematodes. Additionally, comparative pan-genomics with other species of economically important parasitic nematodes have revealed highly open genomes and strong collinearities within the phylum Nematoda.

## Introduction

The barber’s pole worm, *Haemonchus contortus*, is one of the most economically important and common pathogenic nematodes infecting small ruminants worldwide (Laing et al. 2013; Schwarz et al. 2013). Although this parasite can be controlled using anthelmintic drugs, its remarkable natural tendency to develop resistance threatens the global livestock industry. We provide a high-quality draft genome of the anthelmintic-susceptible *H. contortus* NZ_Hco_NP field strain isolated from pasture-grazed New Zealand sheep. *Haemonchuscontortus* that was selected for genome sequencing as part of a wider program investigating the genetic mechanisms that evoke parasite exsheathment.

This blood-feeding strongylid nematode is orally transmitted via contaminated pasture to the host causing anemia, edema, and associated complications often leading to death (Veglia 1915). Currently, control relies heavily on prophylactic drug administration (anthelmintics), but this approach is increasingly under threat due to development of resistance and reduced efficacy of compounds used. Despite the importance of this issue, remarkably little is known regarding the molecular mechanisms of resistance to most anthelmintic drug groups. This is, at least in part, due to: 1) a lack of genomic resources (Consortium 2019); 2) extremely high levels of genetic diversity of worm populations (Gilleard and Redman 2016); 3) limited number of well-characterized anthelmintic resistant isolates (Gilleard 2013); 4) at best only circumstantial and inconsistent list of leading candidate genes (Kotze et al. 2014); 5) tools and techniques with which to study these experimentally challenging organisms (Wit and Gilleard 2017).

Fittingly, the first of any strongylid nematode genome to be sequenced was *H. contortus*; the availability of these whole-genome sequences (Laing et al. 2013; Schwarz et al. 2013), paved the way for substantial advances in several areas of parasite biology, including the genetic machinery involved in life cycle development, reproduction, host–parasite interactions, immunity, and disease. The availability of the *H. contortus* field strain draft genome provides a major resource for future elucidation of the genetic characteristics correlated with its susceptibility, control of life cycle stages and eventually development of next-generation interventions (drugs, vaccines, and diagnostic tests) against *H. contortus* and related nematodes.

The chromosome-level assembly of the draft NZ_Hco_NP genome is 465 Mb in size and encodes 22,341 protein-coding genes (PCGs). In terms of completeness, the genome assembly and associated gene predictions are of similar quality to the two recently published *H. contortus* genomes (Laing et al. 2013; Schwarz et al. 2013). Here, we also describe the complete mitochondrial (mt) genome of NZ_Hco_NP and explore its evolutionary relationships against the complete mt genomes for all 41 nematode species or isolates currently available.

## Materials and Methods

### Production, Procurement, and Electron Microscopy of *H. contortus*

All experimental procedures used in generating the parasite material for this study were approved by AgResearch’s Grasslands Animal Ethics Committee under the Animal Welfare Act 1999 in New Zealand (AEC application number 13928). Adult *H. contortus* were recovered from sheep infected with pure strains of parasites by migration from the abomasal contents gelled in agar (Van Wyk and Gerber 1978). Briefly, abomasal contents were mixed 1:1 with 1.8% bacteriological agar (DIFCO Laboratories) and the solidified agar blocks incubated at 37 °C in a physiological saline (0.9% NaCl solution) bath. Clumps of parasites were removed from the saline soon after emergence, adult male parasites were separated and placed in phosphate buffered saline (1× PBS, 137 mM NaCl, 2.7 mM KCl, 8 mM Na_2_HPO_4_, and 2 mM KH_2_PO_4_, pH 7.4) buffer. Ten male parasites were placed in a 1.8 ml cryogenic vial containing 1 ml of 1× PBS, snap-frozen, and stored at −80 °C until required.

Scanning electron microscopy (SEM) was performed as previously described (Palevich et al. 2017; Palevich, Kelly, et al., 2019). Briefly, cryopreserved worms were gently spun, washed 3× in PBS, and fixed in SEM primary fixative (3% glutaraldehyde, 2% formaldehyde in 0.1 M Phosphate Buffer pH 7.2) for 2 days at room temperature. Samples were dehydrated in a graded ethanol series, that is, 25%, 50%, 75%, and 95% for 10–15 min each and 2× in 100% ethanol for 1 h, then Critical Point (CP) dried using liquid CO_2_ and mounted onto an aluminium specimen support stub using double-sided adhesive tape. Samples were sputter coated with gold (200 sec) and observed using a FEI Quanta 200 Environmental Scanning Electron Microscope (SEM) with energy dispersive X-ray spectroscopy (EDAX) module.

### Preparation of Genomic DNA for Whole-Genome Sequencing

High-molecular weight genomic DNA was isolated from multiple *H. contortus* adult males (anthelmintic-susceptible field strain, New Zealand). Each worm sample was thawed and suspended in 1.5 ml of lysis solution (100 mM NaCl, 100 mM Tris–HCl [pH 8.5], 50 mM EDTA [pH 7.5], 1% SDS, 2% β-mercaptoethanol, and 20 mg/ml proteinase K). After incubation at 55 °C for 3 h with gentle mixing, 10 μl of 100 mg/ml RNase A (QIAgen, Hilden, Germany) was added and the lysate incubated for 1 h at 37 °C. The RNase A was heat inactivated with a 15 min incubation at 70 °C, then placed on ice to cool. DNA was then extracted from the lysate with three successive equal volumes of phenol/chloroform/isoamyl alcohol (25: 24: 1) and once with chloroform/isoamyl alcohol (24: 1). The aqueous layer from the final extraction was transferred to a fresh Eppendorf tube and precipitated with equal volume of isopropanol and 1/10 volume 3 M sodium acetate (pH 5.2), mixed, and placed at −80 °C for 1 h. The DNA was pelleted by centrifugation, washed twice in 70% ethanol (4 °C), briefly air-dried, resuspended in 100 μl TE buffer (10 mM Tris–HCl, 1 mM EDTA, pH 7.5), and stored at −80 °C. The specificity of genomic DNA was verified by automated sanger sequencing of the second internal transcribed spacer (ITS-2) of nuclear ribosomal DNA following PCR amplification from genomic DNA (Bisset et al. 2014). Total DNA amounts were determined using a NanoDrop ND-1000 (Thermo Scientific Inc.) and a Qubit Fluorometer dsDNA BR Kit (Invitrogen), in accordance with the manufacturer’s instructions. Genomic DNA integrity was verified by agarose gel electrophoresis and using a 2000 BioAnalyzer (Agilent).

### Genome Sequencing, Assembly, Gene Calling, and Annotation Comparison

*Haemonchus contortus* NZ_Hco_NP was selected for genome sequencing as a representative of the anthelmintic-susceptible NZ field strain (BioProject ID: PRJNA517503). Illumina TruSeq DNA libraries were constructed from the extracted DNA and whole genome shotgun paired-end (PE) libraries with 350 bp inserts were sequenced on the Illumina HiSeq2500 platform (Macrogen, Korea). A further sample was sequenced using third-generation single-molecule, real-time (SMRT) long-read sequencing technology from Pacific Biosciences (PacBio). These sequencing technologies provided 135- and 10-fold sequencing coverage, respectively (table 1). A de novo hybrid assembly was generated using SPAdes (Bankevich et al. 2012). To improve scaffolding, Chromosomer version 0.1.4a (Tamazian et al. 2016) was used to map contigs that were over 1,000 bp, had nematode protein matches using DIAMOND version 0.9.24 (Buchfink et al. 2015) BlastX or had mRNA-seq reads aligned using STAR version 2.6.1b (Dobin et al. 2013) against the publicly available *H. contortus* draft genomes (Laing et al. 2013; Schwarz et al. 2013). The mitochondrial genome of *H. contortus* NZ_Hco_NP (Palevich, Maclean, Baten, et al. 2019), was assembled de novo based on the Illumina short reads using NOVOPlasy v3.1 (Dierckxsens et al. 2017).

**Table 1:** Comparison of Assembly and Annotation Statistics for the *Haemonchus contortus* NZ_Hco_NP, MHco3 (ISE), and McMaster (HCON_v4) Genomes

	NZ_Hco_NP (v1.0)	MHco3 (ISE)	McMaster (HCON_v4)
Status	High-quality draft	Complete	Draft
Isolation source	Ovine abomasum	Ovine abomasum	Ovine abomasum
Genome project information		
BioSample ID	PRJNA517503	SAMEA2272274	SAMN02251403
BioProject ID	SAMN10834694	PRJEB506	PRJNA205202
Assembly method	SPAdes/NOVOPlasty	Celera/Velvet	Velvet/SOAPdenovo
Genome coverage	150×	100×	185.0×
Sequencing technology	Illumina and PacBio	Illumina and PacBio	Illumina
Scaffold statistics	
Assembled genome size (Mb)	465	283	320
Scaffold count	7	7	14,388
Longest scaffold	93.2 Mb	51.8 Mb	346 kb
Scaffold N50	85.0 Mb	47.4 Mb	56.3 kb
Scaffold N90	78.3 Mb	43.6 Mb	13.1 kb
Number of contigs	16,108	192	55,249
Contig N50	3 kb	3.8 Mb	13 kb
Assembly base composition		
DNA GC (%)	42.1	43.1	42.4
DNA AT (%)	54.2	54.9	51.2
DNA N (%)	3.7	2.0	6.4
Gene model and genome assembly completeness			
Total coding genes	22,341	19,473	23,610
BUSCO: C:P:M (%)^a^	79.2:8.6:12.2	86.5:6.7:6.8	59.8:10.5:29.4
Reference	This report	Laing et al. (2013)	Schwarz et al. (2013)

a C, P, and M refer to fully represented, partially represented, and missing BUSCO genes (Simão et al. 2015).

Repeat sequences were identified and masked using RepeatMasker version 4.0.8 (Tarailo‐Graovac and Chen 2009). Noncoding RNAs, such as microRNAs, were identified by sequence homology search of the Rfam database (Griffiths-Jones et al. 2004). PCGs were predicted using a combination of ab initio programs Snap (Korf 2004), Fgenesh (Solovyev et al. 2006), and the evidence-based predictor Augustus (Stanke et al. 2006) using the annotation pipeline tool Maker (version 2.26) (Cantarel et al. 2008) which aligns mRNA, EST (Hoekstra et al. 2000), and protein information evidence to revise the predicted gene structures. A consensus gene set from the above prediction algorithms was generated, using a previously described, logical, hierarchical approach (Tang et al. 2014).

Genes of interest were annotated using DIAMOND version 0.9.24 BlastP searches against the nematode NCBI reference sequence protein database, KEGG (Kyoto Encyclopedia of Genes and Genomes), and SwissProt databases. Functional domains and Gene Ontology (GO) terms were assigned using InterProScan (5.26–65.0) (Ashburner et al. 2000; Jones et al. 2014). Proteins with signal peptides and transmembrane topology were identified using TMHMM version 2.0 (Krogh et al. 2001) and SignalP version 4.1 (Petersen et al. 2011). CEGMA (Core Eukaryotic Genes Mapping Approach, version 2.4) (Parra et al. 2007) and BUSCO (Benchmarking Universal Single-Copy Orthologs, version 3.0.2) (Simão et al. 2015) were used to assess the completeness of the genome without excluding partial matches. The model nematode *Caenorhabditis elegans* N2 strain genome assembly (WBcel235, GenBank accession GCA_000002985.3) was used for annotation and genome comparison (Kamath et al. 2003). Whole-genome alignments between *H. contortus* NZ_Hco_NP and *C. elegans* N2 were performed using SyMAP version 4.2 (Soderlund et al. 2011), run with the default parameters.

## Results and Discussion

### Genome Assembly and Properties

To sequence the genome of *H. contortus* NZ_Hco_NP, a hybrid strategy using long-read PacBio and short-read Illumina technologies based on 598 million paired-end (PE) and 328,750 reads was applied (supplementary table S3, Supplementary Material online). The genome consists of six chromosomes (465 Mb) and a mitochondrial genome (14,001 kb), with an overall %G + C content of 42.1% (table 1). Additional characteristics and genome project information are shown in table 1 and supplementary table S1, Supplementary Material online. A chromosome-level assembly with high coverage of ∼150× was achieved using insert sizes that ranged between 350 bp (Illumina HiSeq2500) and 20 kb (Pacbio SMRT [Sequel]). In total, 2.9 Gb of trimmed and filtered sequence data were retained for the reported assembly (supplementary table S3, Supplementary Material online), with the estimated genome size in agreement with the initial 453-Mb *H. contortus* assembly previously reported (Schwarz et al. 2013). The genome is highly heterozygous where unclosed gaps (Ns) comprise 3.7% of the genome sequence (table 1). The NZ_Hco_NP genome assembly statistics (table 1) are close to the McMaster (HCON_v4) version (Schwarz et al. 2013) and show better contiguity than the MHco3 (ISE) genome assembly (Laing et al. 2013).

Mitogenomes of parasitic nematodes evolve at a much slower rate than the corresponding nuclear genomes (Hu et al. 2003), possibly reflecting DNA repair and due to variations among individual worms of the population (Hu and Gasser 2006). The complete mitogenome of *H. contortus* NZ_Hco_NP was assembled and gene order, sizes (usually 13.6–14.3 kb) and all common organization features are relatively consistent among the published nematode mitogenomes (Hu and Gasser 2006; Palevich, Maclean, Baten, et al. 2019; Palevich, Maclean, Mitreva, et al. 2019). At the strain level, nucleotide dissimilarity in the *H. contortus* NZ_Hco_NP mitogenome was remarkably high compared with the McMaster (HCON_v4) and MHco3 (ISE) (Palevich, Maclean, Baten, et al. 2019; Palevich, Maclean, Mitreva, et al. 2019). Of the other Strongylida species for which complete mitogenomes are available, *H. contortus* NZ_Hco_NP formed a monophyletic cluster with the remaining Trichostrongylidae species, which then formed a sister clade with the Strongylidae family. The complete NZ_Hco_NP mitogenome is the most divergent among the *H. contortus* strains possibly due to sequencing errors that resulted in assembly artifacts (Palevich, Maclean, Baten, et al. 2019; Palevich, Maclean, Mitreva, et al. 2019). In order to elucidate the maximal levels of divergence within the genus and improve the phylogenetic resolution within the phylum Nematoda, future efforts should focus on the generation of complete mitogenomes across all nematode species, especially for different strains and isolates.

### Genome Annotation

Helminth genome projects identify between 10,000 and 20,000 PCGs per helminth genome, of which approximately half are unknown with respect to functionality (Palevich et al. 2018). Ab initio gene prediction resulted in a total of 22,341 PCGs annotated in *H. contortus* NZ_Hco_NP (table 1). A putative function was assigned to 20,876 PCGs based on the invertebrate NR database, while 16,265 PCGs were annotated as hypothetical proteins or proteins of unknown function. In total, only 4,808 (21.52%) genes have clear homology to proteins in the Swiss-Prot database, 33.46% and 18.13% of annotated genes have well-defined PFAM and RFAM protein domains, respectively, and KEGG numbers were assigned to 18.08% of genes. In contrast, 14,303 (64.02%) and 12,357 (55.31%) of annotated genes have identified InterProScan and RefSeq protein domain hits, respectively. In total, the annotated genic region comprises 9% of the genome with an average of four exons and mean transcript length of 666 bp per gene, respectively, typical of helminth genomes (Consortium 2019).

### Quality Assessment

The CEGMA and BUSCO pipelines were used to assess the NZ_Hco_NP (v1.0) annotation completeness of the conserved core gene set within the assembly. Due to the improved searching algorithm of BUSCO (v3) tool that also utilizes a larger metazoan gene set (978) than CEGMA (248 core eukaryotic genes), only the BUSCO results are reported (table 1). The BUSCO scores revealed a high level of completeness (88%) from the chromosome-level assembly, which is consistent with the CEGMA results. These statistics indicate that the *H. contortus* NZ_Hco_NP (v1.0) genome data outperformed the McMaster (HCON_v4) assembly and is comparable to the updated MHco3 (ISE) genome. The quality of the gene model data set was assessed using various databases (KEGG, InterPro, and GO) and was further manually curated using the Swiss-Prot protein database (supplementary table S4, Supplementary Material online). By focusing on target coverage only as opposed to query coverage, we accounted for potential inaccuracies associated with the lengths of proteins that were typically predicted to have unknown functions. The overall target protein coverage and gene model of NZ_Hco_NP (v1.0) is of considerably higher quality than the MHco3 (ISE) model, and similar to the McMaster (HCON_v4) proteins at the high coverage end.

### Genome Comparison

**Fig. 1.**
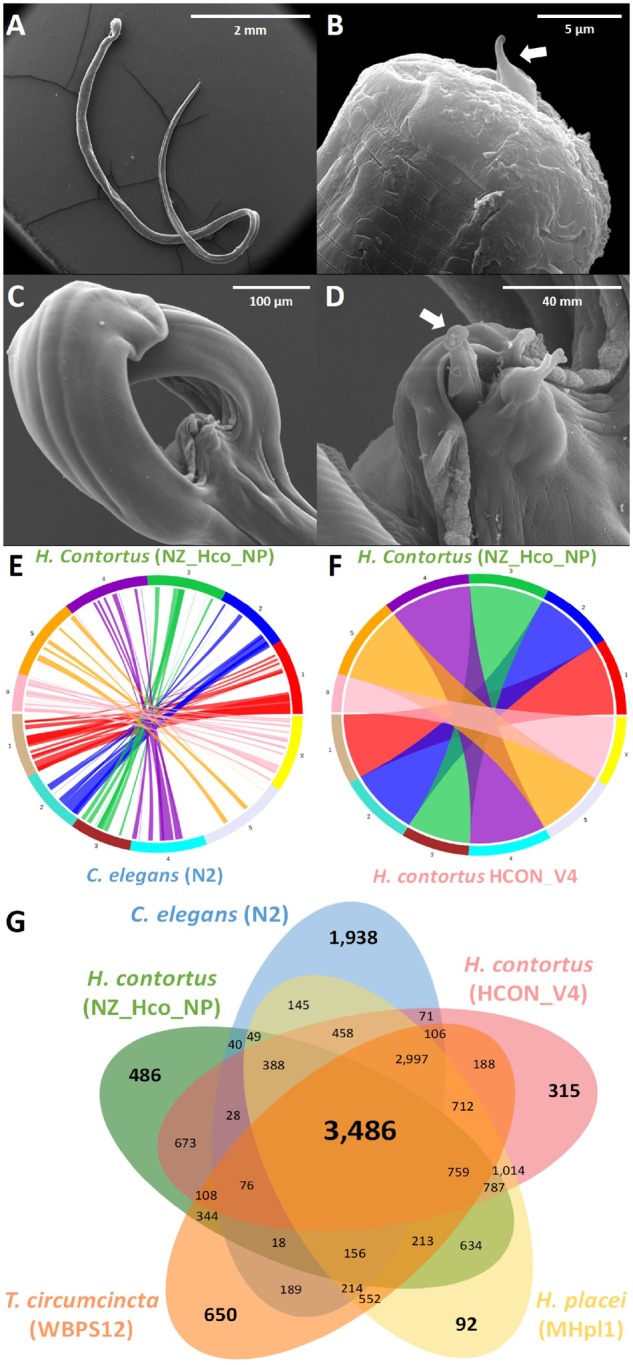
—(*A–D*) Scanning electron micrographs of adult male *Haemonchus contortus* NZ_Hco_NP. (*A*) Entire adult male; (*B*) mouth or anterior end (head) showing the tooth/lancet of buccal cavity (arrow), (*C*) tail or posterior end showing the bursal rays; and (*D*) tip of spicule showing the barb at distal end (arrow). (*E* and *F*) Circos plots showing the syntenic relationships between the six chromosomes of *H. contortus* NZ_Hco_NP with *Caenorhabditis elegans* N2 (*E*) and *H. contortus* HCON_V4 (*F*). Each colored line crossing the circle represents a unique alignment with chromosomes ordered to maximize collinearity among the genomes. (*G*) Venn diagram showing the distribution of shared gene families among the major related genomes of the phylum Nematoda. All *H. contortus* NZ_Hco_NP scaffolds with at least five one-to-one orthologs shared between the two species were compared.

Despite the 4-fold *C. elegans* N2 and near 2-fold *H. contortus* McMaster (HCON_v4) differences in genome size and ∼350 Myr of evolution within the phylum Nematoda, a remarkable degree of collinearity has been observed among these species by comparative genetic mapping (fig. 1*E* and *F*). A comparison of the *H. contortus* NZ_Hco_NP (v1.0) genome with the draft genome of *H. contortus* MHco3 (ISE) (Laing et al. 2013) and the complete *H. contortus* McMaster (HCON_v4) (Schwarz et al. 2013) genome is shown in table 1. Comparative pan-genomics of the economically important parasitic nematodes *H. contortus* (NZ_Hco_NP and HCON_v4), *Haemonchusplacei* (MHpl1), *Teladorsagiacircumcincta* (WBPS12), as well as *C. elegans* (N2), revealed highly open genomes and a strong correlation of orthologous genes among the phylum Nematoda. Most of the predicted *H. contortus* NZ_Hco_NP genes were found to have homologs (BlastP e-value cut-off 10^−5^) in other nematodes (17,950; 80.4%), also largely represented by sets of orthologs (16,875; 75.5%) and 1,075 single-copy genes (fig. 1*G*). In total, 3,486 core genes were found to be orthologous among the five nematode genomes compared, with 759 being shared with at least one other species of nematode except for *C. elegans* (fig. 1*G*). In contrast, only 486 genes (2.2%) were found to be unique to *H. contortus* NZ_Hco_NP relative to the others. Of note, were the 1,543 genes identified to be exclusive to all three parasitic nematode species (≤10^−5^). Of the entire *H. contortus* NZ_Hco_NP gene set, 4,040 genes had an ortholog (≤10^−5^) linked to 1 of 291 known KEGG biological pathways (supplementary table S4, Supplementary Material online). Genomic comparisons with other species within the phylum Nematoda have revealed strong collinearities that will greatly facilitate the isolation of important genes involved in larval development and our understanding of genome evolution. The genome sequence of *H. contortus* NZ_Hco_NP presented here is consistent with the genome architecture of other sequenced *Haemonchus* species and is a valuable resource for future studies regarding population genetic structures of parasitic nematodes.

## Supplementary Material

Supplementary data are available at *Genome Biology and Evolution* online.

## Supplementary Material

Supplementary_Matrial_evz141

## Notes

**Data deposition**: This project has been deposited in Genbank under accession numbers CP035800, CP035805, CP035801, CP035802, CP035804, CP035803 and CP035799.
